# Nutritional value and *in situ* degradability of fruit-vegetable byproducts and their feeding effects on performance of growing Hanwoo steers

**DOI:** 10.5713/ajas.19.0743

**Published:** 2020-02-25

**Authors:** Keun Hong Song, Jun Sik Woo, Ju Ri Kim, Gyeong Lim Ryu, Youl Chang Baek, Young Kyoon Oh, Wan Sup Kwak, Keun Kyu Park

**Affiliations:** 1 Department of Animal Science and Technology, Konkuk University, Seoul 05029, Korea; 2 National Institute of Animal Science, Rural Development Administration, Wanju 55365, Korea; 3 College of Medical Life Sciences, Konkuk University, Chungju 27478, Korea

**Keywords:** Beef Cattle, Steer, Performance, Total Mixed Ration (TMR), Fruit Byproduct, Vegetable Byproduct

## Abstract

**Objective:**

This study was conducted to evaluate nutritional value and *in situ* degradability of fruit-vegetable byproducts and their feeding effects on performance of growing Hanwoo steers.

**Methods:**

Nutritional value and *in situ* degradability of cabbage, Chinese cabbage and fruit-vegetable byproducts were assessed. *In vivo* feeding trial was also performed for 12 weeks. Thirty-six growing steers were randomly allocated into three groups according to body weight (BW) and age in 12 pens (4 replications/treatment) and assigned to one of the three dietary treatments: control (byproduct 0%), FV-B (fruit-vegetable byproduct 20%), and CA-B (cabbage peel 15% plus Chinese cabbage peel 15%, total byproduct 30%).

**Results:**

The crude protein contents of cabbage, Chinese cabbage and fruit-vegetable byproducts were 18.69%, 20.20%, and 10.07%, respectively. Concentrations of neutral detergent fiber (NDF) were higher in cabbage (22.31%) and Chinese cabbage (28.83%) than fruit-vegetable (13.94%). Higher concentrations of non-fiber carbohydrate were observed for fruit-vegetable (66.72%) than cabbage (44.93%) and Chinese cabbage byproducts (24.69%). The effective degradability (ED) of both dry matter (DM) and NDF for fruit-vegetable byproduct (DM, 84.69%; NDF, 85.62%) was higher (p<0.05) than cabbage (DM, 68.47%; NDF, 55.97%) and Chinese cabbage byproducts (DM, 68.09%; NDF, 54.22%). The DM intake was not different among treatments because the amount of feed was kept constant according to the BW of growing steers to prevent overweight during the growing period. The average daily gain during the whole experimental period was not different among treatments (1.26, 1.25, and 1.34 kg/d for control, FV-B, and CA-B). The ED of both DM and NDF degradability of the total mixed ration (TMR) diets were very similar among treatments. Feed conversion ratio during the whole period showed no significant difference among treatments.

**Conclusion:**

This study demonstrates that fruit-vegetable and cabbage byproducts up to 20% and 30% (as fed basis), respectively can be included in TMR diets for growing beef cattle.

## INTRODUCTION

Vast amount of wastes is generated worldwide during the production and processing of fruits and vegetables. Porat et al [1] summarized that 45% and 55% of all fruits and vegetables are lost and not consumed in the world. In Korea, Chinese cabbage is the most important vegetable to make kimchi, a traditional fermented food. The total annual production of Chinese cabbage and cabbage from 2011 to 2017 in Korea was approximately two and a half million tons, and its value is estimated as the fourth largest for cabbage in the world [2]. During the processes of production, postharvest storage, transportation, and further processing, up to 30% of the total production is discarded as waste [3]. In the wholesale market, which is a stage before distribution to retailers or intermediate merchants, the disposal rate is also very high. For instance, Chinese cabbage occupied 11.1% (449,908 ton/yr) and cabbage 4.5% (182,175 ton/yr) of the total vegetables traded within the biggest public wholesale market in Seoul, Korea. The amount of wastes generated from fruit and vegetable in this market was 30,076 kg/d on average, and waste productions of Chinese cabbage and cabbage estimated 14,831 and 1,483 kg/d, respectively [4].

The most common method to treat these wastes is throwing them into landfills, which can result in many unpleasant environmental consequences. Although various methods have been proposed to dispose or recycle of fruit and vegetable wastes in wholesale markets and packing houses, their utilization as a feed ingredient for ruminant diets seems to be the most eco-friendly and value-added option [1]. Since these waste resources contain high amounts of moisture, manufacturing high moisture total mixed ration (TMR), blending all feedstuffs into a complete ration that does not require drying process could be the most feasible and economical way.

In addition, fruit and vegetable wastes, having high amount of moisture as well as soluble carbohydrates, can readily be decomposed by putrefying microorganisms, and promote secondary fermentation along with offensive odor. Recently, our earlier investigations revealed that using 0.6% of sodium metabisulfite (SMB; Na_2_S_2_O_5_) was highly effective in maintaining the integrity and freshness of fruit and vegetable wastes by suppressing microbial proliferation and preserving the nutrient components [5,6]. Silage fermentation without SMB was not effective for preventing the deterioration and mold growth, which resulted in the rapid depletion of sugars and dry matter (DM) [6]. Therefore, the aim of this study was to evaluate nutritional value and rumen degradability of cabbage, Chinese cabbage and fruit-vegetable residues, and to examine feeding effects of these byproducts on performance of growing Hanwoo steers to determine its potential use as feedstuff for bovine feeding, which would contribute to minimizing its environmental impact.

## MATERIALS AND METHODS

### Animal care

The animal use and protocols employed during the research were reviewed and approved by the Institutional Animal Care and Use Committee at Konkuk University (Approval number: KU18129).

### Fruit-vegetable byproducts

The fruit-vegetable byproduct was obtained from a wholesale fruit and vegetable distribution center (E-mart Fresh Center; Icheon, Gyeonggi, Korea), containing various types of fruits and vegetables (orange, apple, tomatoes, onion, bok choy, bell pepper, etc.). These byproducts tend to putrefy quickly because of their high content of moisture and simple carbohydrates. Thus, these byproducts were milled into 30 to 40 mm pieces, treated with 6 g/kg of SMB, and stored in 110-L polyvinyl chloride bins to extend the length of storage. The level of SMB was determined by the results from Ahmadi et al [5].

Another byproduct consisting of cabbage and Chinese cabbage peels was produced from a traditional wholesale market in Seoul, Korea. These vegetables are particularly in large demand; thus, their byproducts occur constantly throughout the year. The vegetable byproducts were added in TMR diets as a raw material without pretreatment.

### Chemical analysis

All samples of byproducts and TMR diets for chemical analysis were dried at forced-air oven connected to the sucking fan at 60°C for over 24 hours and ground through 2 mm screen using Wiley mill. Samples were analyzed for DM [7] (AOAC official method 930.15), crude protein (CP) [7] (AOAC official methods 930.15 and 990.03), fat [8] (AOAC official methods 930.15 and 2003.05), lignin [9], acid detergent fiber (ADF) [7] (AOAC official methods 930.15 and 973.18), neutral detergent fiber (NDF) [10], and ash [7] (AOAC official method 942.05).

### *In situ* dry matter degradability

Two Holstein cows (405±15.4 kg) fitted with ruminal cannula were fed 4 kg of tall fescue and 3 kg of a formulated concentrate mix, divided into equal portions and offered daily at 09:00 and 18:00. The animals had free access to fresh water and mineral block. Rumen degradability of byproducts was determined using nylon bags (10×20 cm and 50 μm of pore size; Ankom Technology, Macedon, NY, USA) and filled with approximately 4 g of sample. The ratio of sample size to bag surface area was equal to 10 mg/cm^2^, which is within the range (10 to 20 mg/cm^2^) recommended by the previous report [11]. Triplicate bags were prepared in each animal for each incubation time.

Immediately after morning feeding, the bags containing the sample from fruit-vegetable, cabbage or Chinese cabbage byproduct were incubated in the ruminal ventral sac for 0, 4, 8, 12, 24, 48, 72, 96, and 120 h. The time of entry into the rumen for each incubation time was adjusted to minimize the number of nylon bags staying in a rumen, instead of the ‘all in/gradual out’ [11] or the ‘gradual addition/all out’ schedule [12], thus maximum number of bags in the rumen at any one time was less than 15. Thus, this method may be called gradual addition/gradual out. After incubation, bags were immediately washed in cold tap water until the rinsed water became clear, were dried at 65°C in a drying oven for 48 h and cooled in a desiccator and weighed. Bag residues of the same samples were then pooled for each time of incubation and analyzed for NDF with the analytical procedure [10].

The degradability of DM from nylon bag was fitted to the exponential model and effective degradability (ED) were calculated as follows [13]:

$$\begin{array}{l} \text{P}=\text{a}+\text{b}(1-{\text{e}}^{-\text{ct}})\\ \text{ED}=\text{a}+\text{b}\times \text{c}/(\text{c}+\text{k}) \end{array}$$

Where, P is the DM degradability at time t, ‘a’ rapidly degradable fraction, ‘b’ the insoluble but potentially degradable fraction, ‘c’ constant for b fraction, ‘t’ rumen suspension time and ‘k’ outflow rates in rumen, assuming an outflow rate (k) of 0.04 per hour.

### Animal trial

Thirty-six growing steers (initial body weight [BW] 287±89 kg, 9.1±2.7 months) were randomly allocated into three experimental groups according to BW and age, consisting of three animals per pen in a total of 12 pens (4 replications/treatment). After an adaptation period of 2 weeks, animals were fed the following experimental dietary treatments administered according to groups for 12 weeks. The treatments were control (byproduct 0%), FV-B (fruit-vegetable byproduct 20%, as fed basis), and CA-B (total byproduct 30% including cabbage peel 15% plus Chinese cabbage peel 15%, as fed basis). TMR diets were formulated based on the Korean Feeding Standard for growing Hanwoo steers [14] to meet energy and protein requirements (total digestible nutrients [TDN] 73% and CP 16%). Ingredients and chemical composition of the experimental diets are presented in Table 1 and 2, respectively. In addition, TMR diets fed were measured for *in situ* DM and NDF degradability. The method was carried out in the same manner as mentioned above.

Feeds were offered twice equally at 08:00 and 17:00 h daily. Animals could access fresh water and mineral block without any restriction during the whole period. Steers were weighed at the initial stage of the experiment and every 4 weeks (1st period, 0 to 4 weeks; 2nd period, 5 to 8 weeks; 3rd period, 9 to 12 weeks) before morning feeding. The DM intake (DMI) was measured every week for calculating feed conversion ratio (FCR) (feed/gain).

### Calculations and statistical analysis

Non-fiber carbohydrate (NFC), TDN, and FCR value of experimental feeds were calculated as follows: i) NFC calculated by difference: 100−[CP+(NDF−NDICP)+EE+ash] [15]. ii) TDN = 0.93×CP+0.92×(1+EE−ash−CP−NDF)+0.75×(NDF− ADL)×(1−ADL^2/3^/(NDF)^2/3^) [16]. iii) FCR = dry matter intake (kg)/gain (kg). Where, NDICP, neutral detergent insoluble crude protein; EE, ether extract; ADL, acid detergent lignin.

Data obtained from *in situ* degradability and *in vivo* trial (BW, average daily gain [ADG], DMI, and FCR) were subjected to statistical analysis using the MIXED procedure of SAS (SAS Inst. Inc., Cary, NC, USA). For the *in situ* trial, the model included byproducts as the fixed effect and animals as the random effect. For the *in vivo* trial, the model included diets as the fixed effect and initial BW as the random effect. The pen was considered as the animal unit. The LSMEANS was used to calculate the mean values of treatments. Least squares means among treatments were compared using the probability of difference (PDIFF) option when the treatment effect was significant. A p-value used for the determination of statistical significance was 0.05.

## RESULTS AND DISCUSSION

### Chemical analysis

The chemical composition of byproducts is reported in Table 3. As expected, moisture concentrations of all byproducts were high, containing 90.40%, 93.75%, and 85.19% for cabbage, Chinese cabbage, and fruit-vegetable byproducts, respectively. The high moisture content of leafy vegetables such as cabbage and Chinese cabbage has been known to negatively correlate with DMI [17,18]. Therefore, manufacturing TMR from these byproducts is necessary not only to reduce the moisture content but also to ensure economic feasibility in order to facilitate their inclusion in animal diets.

The DM of cabbage byproducts was relatively rich in protein. The CP concentrations of cabbage and Chinese cabbage were higher than that of fruit-vegetable. The variation in the CP content of each byproduct was also very low, having low standard deviation (SD). The CP content of cabbage was similar to the data (20.4%) from Wadhwa et al [19] who reported the nutrient composition of cabbage peels. The CP values of fruit-vegetable found in this study were similar to those of Angulo et al [20] and Esteban et al [21], who found that fruit-vegetable byproduct contained 10% to 12%.

Relatively higher NDF concentrations were observed in cabbage and Chinese cabbage than in fruit-vegetable, having 22.31%, 28.83%, and 13.94%, respectively. Fiber fractions of byproducts were more variable than other nutrient composition, having relatively higher SD values. For instance, NDF content ranged from 14.25% to 27.07% for cabbage and 25.06% to 34.20% for Chinese cabbage byproducts. The present results were comparable to those from Ngu and Ledin [18], who reported that NDF of cabbage and Chinese cabbage were 27.9% and 25.3%, respectively. Higher fiber fractions of cabbage peels were reported by Wadhwa et al [19], containing NDF 34.0%, ADF 23.0%, ADL 4.22%. The variation in chemical composition can be attributed to the plant species, season, climate, and agronomic practices adapted in different regions [18]. Esteban et al [21] showed that crude fiber percentage of fruit-vegetable residue was 13% which was higher than the values found in this study. However, Angulo et al [20] reported even higher fiber fractions of fruit-vegetable (NDF 36.6%, ADF 29.6%) which varied by sampling period. Concentrations of ADL were relatively low, containing 1.90% for cabbage, 4.63% for Chinese cabbage and 4.62% for fruit-vegetable.

Higher amount of NFC was noted for fruit-vegetable (66.72%) than cabbage (44.93%) and Chinese cabbage byproducts (24.69%). This was a part of the reason why SMB treatment was needed for fruit-vegetable, though the storage period was less than 5 days before manufacturing of TMR. The ash concentration of Chinese cabbage byproduct was very high (29.15%), thereby lowering the concentration of NFC and TDN. Thus, major minerals and heavy metals were analyzed for all three byproducts to determine the presence of harmful minerals. However, the levels of heavy metals were negligible and far below the acceptance criteria of the Korea Ministry of Agriculture, Food and Rural Affairs (data not shown).

#### In situ DM and NDF degradability of byproducts

The *in situ* rumen DM degradability of byproducts is shown in Table 4. At 0 h of incubation, the degradability of cabbage, Chinese cabbage and fruit-vegetable exceeded 30%, and higher degradability was noticed (p<0.05) for fruit-vegetable (42.25%) than for cabbage (32.64%) and Chinese cabbage byproducts (33.34%). After 4 h of incubation, the degradability for these byproducts reached more than 50%. Similar to these results, Angulo et al [20] found that more than 50% of fruit-vegetable was degraded at 2 h, and more than 80% was degraded at 24 h for all periods. These results are mainly due to the highly soluble sugar and protein fractions of these byproducts. According to Wadhwa et al [19], leaves from cauliflower and cabbage have a high concentration of total sugars (18.6% and 20.6%, dry matter basis [DMB]) and water soluble protein fractions constituted the major portion (54% to 62%) of true protein of these vegetables.

The degradability of both cabbage and Chinese cabbage byproducts slightly increased from 4 to 12 h, increased exponentially from 12 to 24 h, then plateaued after 48 h. Meanwhile, the degradation pattern of fruit-vegetable steadily increased until 24 h and then reached plateau. Although the difference was not large, the degradability was the highest in fruit-vegetable, followed by cabbage, and the lowest in Chinese cabbage byproducts during the whole incubation times (p<0.05). The ED of fruit-vegetable byproduct (84.69%) were higher (p< 0.05) than those of cabbage (68.47%) and Chinese cabbage byproducts (68.09%). Similarly, the ED of residues containing half fruit and half vegetable was approximately 85% and not differed by the sampling periods [23]. Rumen DM degradability of tomato pulp, apple pulp and cauliflower were 47.1%, 89.1%, and 93.8%, respectively, suggesting that fruit-vegetable was more degradable than tomato pulp and more like fruit degradation [24].

The *in situ* NDF degradability of byproducts is presented in Table 5. At the initial 0 h incubation time, the NDF degradability of cabbage, Chinese cabbage and fruit-vegetable was very high, being more than 46%. Then, the pattern of NDF degradation of cabbage and Chinese byproducts was similar to that of DM, slowly increasing up to 12 h, significantly increased between 12 and 24 h, and reached plateau after 48 h. On the other hand, the NDF fraction of fruit-vegetable was degraded exponentially from 12 to 48 h, then reached plateau after 72 h. Overall, the NDF degradability of fruit-vegetable by-product except at 0 h was higher (p<0.05) than those of cabbage and Chinese cabbage by-products during the whole incubation time. The final NDF degradability of both cabbage and Chinese cabbage was approximately 66%, whereas that of fruit-vegetable reached 92%. This difference become more apparent when comparing digestion kinetics parameters. The rapidly degradable fraction ‘a’ of fruit-vegetable by-product (75.18%) was much higher (p<0.05) than cabbage (48.72%) and Chinese cabbage by-products (47.35%). Consequently, The ED of NDF for fruit-vegetable byproduct (85.62%) were higher (p<0.05) than those of cabbage (55.97%) and Chinese cabbage byproducts (54.22%). These results suggest that the NDF fraction of fruit-vegetable byproduct is more degradable than those of cabbage and Chinese cabbage byproducts.

### Animal performance

The BW, DMI, ADG, and FCR of control and byproduct treatments are presented in Table 6. Initial (0 day) BW of control, FV-B and CA-B were 288.8, 310.9, and 282.1 kg, respectively. Final (12 weeks) BW were not different among treatments. In addition, there were no statistical differences among treatments during the middle of experimental days (4 and 8 weeks).

The average DMI during the whole period was not significantly different between treatments throughout the whole experimental period. This was because the ADG at the first period was already above the target gain (<1.0 kg) regardless of dietary treatments and the amount of TMR offered was kept constant according to the BW of steers to prevent overweight during the growing period. All animals were willing to consume more feeds than offered. Excessive daily gains during the growing period may lead to decreased or rejected feed intake at the end of the finishing period, resulting in reduced marbling deposits [14,25]. The ADG at the 1st, 2nd, and 3rd period was not different among treatments, and thus the ADG during the entire experimental period was not different among treatments. According to NIAS [26], recommended levels of CP and TDN in feeds of Hanwoo steers at the growing period (up to 13-month-old) are 16% and 72% to 73%, respectively. The target ADG from 8- (BW 208 kg) to 12-month-old (BW 310 kg) is 0.83 to 0.90 kg. The TMR diet in this experiment was formulated to meet these nutrient requirements while maximizing the amount of by-products added. Consequently, the ADG of all treatments exceeded the value of the feeding standard with same levels of CP and TDN.

The *in situ* rumen DM and NDF degradability of the TMR diets fed to the experimental animals is presented in Table 7 and 8, respectively. The DM degradability of control, FV-B and CA-B at 0 h was approximately 32%, steadily increased to 80% until 48 h, then slightly increased up to 86% thereafter. The digestion parameters such as a and b fractions, ED of DM degradability were not different among treatments. The NDF degradability of these diets showed little changes until 12 h, but greatly increased from 66% to 84% for 12 to 48 h. The ED of both DM and NDF degradability of the TMR diets were almost identical among treatments because TMR diets were formulated at the same level of DM and NDF as well as other nutrients among treatments.

No statistical differences were noted for FCR among treatments during the whole experimental period. Froetschel et al [27] examined grocery byproduct feed (GBP) consisting of fruit, vegetables, and bakery items from large retail stores. Steers were fed 0%, 20%, 40%, and 60% ensiled GBP in a TMR (90.8% DM), being equivalent to 0%, 18.2%, 36.3%, and 54.4% of the ration DM. The highest intakes were observed at the 18.2% and 36.3% GBP inclusion rates. The nutrient composition of the ensiled GBP used was 17.7% DM, 12.3% CP, 14.1% NDF, 6.6% ash, and 85% TDN. In this study, inclusion levels of byproducts were 20% for FV-B (13.2% on DMB with 34% moisture) and 30% for CA-B (19.8% on DMB with 34% moisture). The levels were close to the maximum level based on the feed formulation program [18] because relatively high concentrations of moisture and low TDN of these byproducts limit higher inclusion level in TMR diets in order to meet the requirement of growing Hanwoo cattle [11]. It was concluded that FV-B and CA-B can be used effectively as feed resources in TMR diets for cattle.

## CONCLUSION

The use of FV-B up to 20% and CA-B up to 30% in TMR (as fed basis) did not show any negative effect on the performance of Hanwoo steers in the growing period. Therefore, these byproducts could serve as sources of nutrients for ruminants and can economize the production cost of animals.

## Figures and Tables

**Table 1:** Formula of experimental TMR diets for growing Hanwoo steers (%)

Items	Control1)	FV-B1)	CA-B1)
Corn flake	18.6	18	20
Fruit-vegetable byproduct	-	20	-
Chinese cabbage peel	-	-	15
Cabbage peel	-	-	15
Rice bran	8	7	6
Perilla meal	7	7	6
Lupine	5	6	5.4
Wheat bran	5	2	4
Alfalfa	10	10	10
Timothy	17.8	17	17
Water	27	10.4	-
Vitamin-mineral mix	0.3	0.3	0.3
Limestone	1	1	1
Salt	0.3	0.3	0.3
Total	100	100	100

TMR, total mixed rations (as fed basis).

1) Control, 0% of fruit or vegetable byproduct in TMR; FV-B, 20% of fruit-vegetable byproducts including various types of fruits and vegetables (orange, apple, tomatoes, onion, bok choy, bell pepper, etc.) in TMR; CA-B, 30% of byproducts including Chinese cabbage peels (15%) and cabbage peels (15%) in TMR.

**Table 2:** Chemical composition of experimental TMR diets (DM basis, %)

Items	Control1)	FV-B1)	CA-B1)
Dry matter	63.81	64.39	66.20
Crude protein	16.37	17.22	16.32
Ether extract	5.83	6.17	5.30
Ash	8.08	8.00	8.49
Neutral detergent fiber	40.82	38.08	39.75
Acid detergent fiber	24.35	22.91	22.76
Acid detergent lignin	5.52	5.36	5.31
Neutral detergent insoluble crude protein	4.55	4.34	4.35
Acid detergent insoluble crude protein	1.25	1.05	1.13
Total digestible nutrients	72.08	73.41	71.77

TMR, total mixed rations (as fed basis).

1) Control, 0% of fruit or vegetable byproduct in TMR; FV-B, 20% of fruit-vegetable byproducts including various types of fruits and vegetables (orange, apple, tomatoes, onion, bok choy, bell pepper, etc.) in TMR; CA-B, 30% of byproducts including Chinese cabbage peels (15%) and cabbage peels (15%) in TMR.

**Table 3:** Chemical composition of byproducts (DM basis, %)

Items	Cabbage byproduct1)		Chinese cabbage byproduct1)		Fruit-vegetable byproduct1)	
		
	Mean	SD	Mean	SD	Mean	SD
Dry matter	9.60	0.74	6.25	0.83	14.81	0.49
Crude protein	18.69	0.53	20.20	0.60	10.07	0.33
Ether extract	2.11	0.17	2.04	0.81	2.63	0.26
Crude fiber	13.51	1.42	13.80	1.49	7.59	0.42
Ash	13.67	0.87	29.15	3.85	6.65	0.36
Neutral detergent fiber	22.31	4.67	28.83	3.61	13.94	3.24
Acid detergent fiber	15.73	3.26	17.73	4.29	12.14	0.74
Acid detergent lignin	1.90	0.91	4.63	1.47	4.62	0.40
Neutral detergent insoluble crude protein	2.85	1.01	4.59	1.12	-	-
Acid detergent insoluble crude protein	0.96	0.16	1.19	0.14	-	-
Non-fiber carbohydrate	44.93	5.09	24.69	4.88	66.72	3.28
Total digestible nutrient	73.30	2.62	53.53	5.56	79.53	0.89

DM, dry matter; SD, standard deviation of samples.

1) Cabbage and Chinese cabbage byproducts mainly contain outer peels of the vegetable (n = 7) and fruit-vegetable byproduct contains byproduct from various types of fruits and vegetables (n = 7).

**Table 4:** *In situ* rumen DM degradability and ED of byproducts (%)

Items	Cabbage byproduct1)	Chinese cabbage byproduct1)	Fruit-vegetable byproduct1)	SEM
Time (h)				
0	32.64^a^	33.34^a^	42.25^b^	0.69
4	51.48^a^	49.97^a^	68.35^b^	1.97
8	56.43^b^	51.15^a^	74.03^c^	1.14
12	58.10^b^	53.56^a^	81.58^c^	1.35
24	78.36^a^	83.05^a^	92.72^b^	1.68
48	95.03^b^	90.29^a^	95.61^b^	0.42
72	93.62^b^	90.99^a^	95.79^c^	0.46
96	92.54^b^	90.99^a^	95.01^c^	0.40
120	91.08^a^	90.01^a^	96.10^b^	0.65
Degradation characteristics of DM and ED2)				
a	34.47^b^	26.32^a^	50.20^c^	1.33
b	56.61^b^	63.90^c^	45.24^a^	1.03
c	0.060^a^	0.076^b^	0.129^c^	<0.01
a+b	91.08^a^	90.22^a^	95.44^b^	0.63
ED	68.47^a^	68.09^a^	84.69^b^	0.86

DM, dry matter; ED, effective degradability; SEM, standard error of means.

1) Cabbage and Chinese cabbage byproducts, mainly contain outer peels of the vegetable; Fruit-vegetable byproduct, contains byproduct from various types of fruits and vegetables.

2) a, rapidly degradable fraction (%); b, insoluble fraction but degraded over time in rumen (%); c, constant for b fraction in the exponential equation (fraction/h); a+b, potentially degradable fraction (%); ED (%), effective degradability calculated with outflow rates of 4%, t = 120 h.

a–c Means within a row without a common superscript letter differ at p<0.05.

**Table 5:** *In situ* rumen NDF degradability and ED of byproducts (%)

Items	Cabbage byproduct1)	Chinese cabbage byproduct1)	FV-B1)	SEM
Time (h)				
0	49.94^b^	46.10^a^	47.87^a^	0.46
4	49.87^a^	51.00^a^	53.59^b^	0.64
8	50.37^a^	53.35^ab^	57.06^b^	0.64
12	52.22^a^	56.52^b^	58.46^b^	0.56
24	64.38^a^	65.01^a^	70.01^b^	1.43
48	67.85^a^	66.05^a^	86.34^b^	0.71
72	65.19^a^	64.76^a^	90.55^b^	0.47
96	66.08^a^	66.74^a^	89.23^b^	0.65
120	65.63^a^	66.42^a^	91.52^b^	0.38
Degradation characteristics of NDF and ED2)				
a	48.72^a^	47.35^a^	75.18^b^	1.11
b	16.91	19.07	16.34	1.02
c	0.030^a^	0.025^a^	0.072^b^	0.01
a+b	65.63^a^	66.42^a^	91.52^b^	0.38
ED	55.97^a^	54.22^a^	85.62^b^	0.78

NDF, neutral detergent fiber; ED, effective degradability; SEM, standard error of means.

1) Cabbage and Chinese cabbage byproducts mainly contain outer peels of the vegetable (n = 7) and fruit-vegetable byproduct contains byproduct from various types of fruits and vegetables (n = 7).

2) a, rapidly degradable fraction (%); b, insoluble fraction but degraded over time in rumen (%); c, constant for b fraction in the exponential equation (fraction/h); a+b, potentially degradable fraction (%); ED (%), effective degradability calculated with outflow rates of 4%, t = 120 h.

a,b Means within a row without a common superscript letter differ at p<0.05.

**Table 6:** Effects of feeding byproducts on performance in growing Hanwoo steers

Items	Treatments1)			SEM	p-value

	Control	FV-B	CA-B		
BW (kg)					
0 wk	288.8	310.9	282.1	23.044	0.068
4th wk	335.0	357.9	331.6	24.629	0.109
8th wk	370.7	392.0	359.6	23.749	0.154
12th wk	399.2	411.6	389.9	24.954	0.399
DMI (kg/d)					
1st period2)	6.90	7.67	7.41	0.562	0.382
2nd period	7.16	7.61	7.23	0.674	0.481
3rd period	7.25	7.81	7.28	0.531	0.071
Whole period	7.06	7.63	7.15	0.585	0.180
ADG (kg/d)					
1st period	1.74	1.60	1.69	0.149	0.803
2nd period	1.15	1.28	1.04	0.076	0.152
3rd period	0.87	0.96	1.27	0.156	0.283
Whole period	1.26	1.25	1.34	0.122	0.859
FCR (F/G)					
1st period	4.17	5.02	4.47	0.560	0.553
2nd period	6.47	6.11	7.46	0.756	0.464
3rd period	8.50	7.97	7.48	1.288	0.859
Whole period	5.87	6.36	5.80	0.760	0.756

SEM, standard error of means; BW, body weight; DMI, dry matter intake; ADG, average daily gain; FCR, feed conversion ratio.

1) Control, byproduct 0%; FV-B, fruit-vegetable byproducts 20%; CA-B, Chinese cabbage and cabbage byproducts 30%.

2) 1st period (0 to 4 wk); 2nd period (5 to 8 wk); 3rd period (9 to 12 wk); whole period (0 to 12 wk).

**Table 7:** *In situ* rumen DM degradability and ED of TMR diets (%)

Items	Control1)	FV-B1)	CA-B1)	SEM
Time (h)				
0	31.03	32.77	31.69	0.61
4	36.77	37.47	37.24	0.44
8	40.59	41.31	39.78	0.71
12	53.66	52.14	54.39	1.77
24	69.85	70.46	68.67	1.90
48	80.68	81.02	80.11	0.87
72	82.54	82.34	82.70	0.84
96	83.14	83.68	83.18	0.57
120	85.82	86.06	85.19	0.30
Degradation characteristics of DM and ED2)				
a	38.02	38.32	35.93	0.45
b	47.81	47.75	49.26	0.57
c	0.035	0.035	0.038	0.01
a+b	85.83	86.07	85.19	0.38
ED	60.26	60.69	59.90	0.78

DM, dry matter; ED, effective degradability; TMR, total mixed ration; SEM, standard error of means.

1) Control, byproduct 0%; FV-B, fruit-vegetable byproducts 20%; CA-B, Chinese cabbage and cabbage byproducts 30%.

2) a, rapidly degradable fraction (%); b, insoluble fraction but degraded over time in rumen (%); c, constant for b fraction in the exponential equation (fraction/h); a+b, potentially degradable fraction (%); ED (%), effective degradability calculated with outflow rates of 4%, t = 120 h.

**Table 8:** *In situ* rumen NDF degradability of TMR diets (%)

Items	Control1)	FV-B1)	CA-B1)	SEM
Time (h)				
0	56.40	58.94	62.84	1.44
4	60.43	62.37	63.98	1.72
8	63.78	63.96	65.23	1.68
12	65.17	65.45	68.25	0.98
24	71.45	70.11	70.35	1.68
48	84.05	82.71	83.40	0.96
72	84.71	84.10	83.49	0.73
96	85.60	83.84	86.29	0.42
120	84.60	84.27	85.51	1.16
Degradation characteristics of NDF and ED2)				
a	56.91	58.58	60.02	1.34
b	27.68	25.69	25.49	1.35
c	0.032	0.036	0.044	0.01
a+b	84.60	84.27	85.51	1.16
ED	69.00	70.78	72.81	1.11

NDF, neutral detergent fiber; TMR, total mixed ration; SEM, standard error of means; ED, effective degradability.

1) Control (Byproduct 0%); FV-B (Fruit-vegetable byproducts 20%); CA-B (Chinese cabbage and cabbage byproducts 30%); SEM, standard error of means.

2) a, rapidly degradable fraction (%); b, insoluble fraction but degraded over time in rumen (%); c, constant for b fraction in the exponential equation (fraction/h); a+b, potentially degradable fraction (%); ED (%), effective degradability calculated with outflow rates of 4%, t = 120 h.
